# Supplementary material for: Psychological Status of Men Who Have Sex with Men during COVID-19: An Online Cross-Sectional Study in Western China
Source: Int J Environ Res Public Health. 2023 Jan 11;20(2):1333. doi: 10.3390/ijerph20021333 (PMC9858883; doi:10.3390/ijerph20021333)
Supplement: Supplementary file 1 [file ijerph-20-01333-s001.zip › ijerph-2093681-supplementary.pdf]

Supplementary Table S1.

Univariate analysis of each variable

Variables		Higher risk of depression			Higher risk of neurasthenia			Higher risk of fear		
	Total n (%)	Yes n (%)	No n (%)	p-value	Yes n (%)	No n (%)	p-value	Yes n (%)	No n (%)	p-value
**Demographic characteristics [a]**										
Age				**0.021**			**0.096**			0.889
18~35	248(60.19)	52(74.29)	196(57.31)		40(68.97)	208(58.76)		41(59.42)	207(60.35)	
35~50	142(34.47)	17(24.29)	125(36.55)		18(31.03)	124(35.03)		25(36.23)	117(34.11)	
⩾50	22(5.34)	1(1.43)	21(6.14)		0(0.00)	22(6.21)		3(4.35)	19(5.54)	
Household location				0.245			0.404			**0.089**
Urban	388(94.17)	68(97.14)	320(93.57)		56(96.55)	332(93.79)		68(98.55)	320(93.29)	
Rural	24(5.83)	2(2.86)	22(6.43)		2(3.45)	22(6.21)		1(1.45)	23(6.71)	
Ethnicity				0.867			0.465			0.508
Han ethnicity	373(90.53)	63(90.00)	310(90.64)		51(87.93)	322(90.96)		61(88.41)	312(90.96)	
Minority	39(9.47)	7(10.00)	32(9.36)		7(12.07)	32(9.04)		8(11.59)	31(9.04)	
Educational level				0.622			0.629			0.108
High school or below	61(14.81)	8(11.43)	53(15.50)		9(15.52)	52(14.69)		15(21.74)	46(13.41)	
Vocational school	150(36.41)	28(40.00)	122(35.67)		24(41.38)	126(35.59)		27(39.13)	123(35.86)	
College or above	201(48.79)	34(48.57)	167(48.83)		25(43.10)	176(49.72)		27(39.13)	174(50.73)	
Employment status				0.845			0.306			0.423
Employed	315(74.29)	52(74.29)	263(76.90)		42(72.41)	273(77.12)		49(71.01)	266(77.55)	
Unemployed/Retirement	73(17.72)	13(18.57)	60(17.54)		14(24.14)	59(16.67)		16(23.19)	57(16.62)	
Student	24(5.83)	5(7.14)	19(5.56)		2(3.45)	22(6.21)		4(5.80)	20(5.83)	
Marital status				**0.050**			**0.081**			0.890
Married	68(16.50)	6(8.57)	62(18.13)		5(8.62)	63(17.80)		11(15.94)	57(16.62)	
Unmarried	344(83.50)	64(91.43)	280(81.87)		53(91.38)	291(82.20)		58(84.06)	286(83.38)	
Monthly personal income				0.878			0.576			0.430
1000~5000 RMB	201(48.79)	36(51.43)	165(48.25)		32(55.17)	169(47.74)		38(55.07)	163(47.52)	
5000~10000 RMB	139(33.74)	22(31.43)	117(34.21)		17(29.31)	122(34.46)		22(31.88)	117(34.11)	
⩾10000 RMB	72(17.48)	12(17.14)	60(17.54)		9(15.52)	63(17.80)		9(13.04)	63(18.37)	

**HIV-related characteristics [a]**										
Sexual role				**0.077**			0.381			0.330
Mainly "Top"	182(44.17)	29(41.43)	153(44.74)		22(37.93)	160(45.20)		31(44.93)	151(44.02)	
Both of it	72(17.48)	7(10.00)	65(19.01)		9(15.52)	63(17.80)		8(11.59)	64(18.66)	
Mainly "Bottom"	158(38.35)	34(48.57)	124(36.26)		27(46.55)	131(37.01)		30(43.48)	128(37.32)	
Number of male sexual partners in the last month				0.146			**0.048**			0.966
0	227(55.10)	46(65.71)	181(52.92)		40(68.97)	187(52.82)		39(56.52)	188(54.81)	
1	123(29.85)	16(22.86)	107(31.29)		14(24.14)	109(30.79)		20(28.99)	103(30.03)	
⩾2	62(15.05)	8(11.43)	54(15.79)		4(6.90)	58(16.38)		10(14.49)	52(15.16)	
Self-reported HIV infection status				0.263			0.429			0.273
Positive	38(9.22)	10(2.43)	28(8.19)		8(13.79)	30(8.47)		9(13.04)	29(8.45)	
Negative	358(86.89)	57(81.43)	301(88.01)		48(82.76)	310(87.57)		59(85.51)	299(87.17)	
Unknown	16(3.88)	3(4.29)	13(3.80)		2(3.45)	14(3.95)		1(1.45)	15(4.37)	
**COVID-19 related knowledge [b]**										
I am well informed about the causes of COVID-19				**0.064**			0.155			0.288
Totally disagree	21(5.10)	7(10.00)	14(4.09)		8(13.79)	13(3.67)		8(11.59)	13(3.79)	
Not quite agree	40(9.71)	13(18.57)	27(7.89)		8(13.79)	32(9.04)		8(11.59)	32(9.33)	
Average	135(32.77)	17(24.29)	118(34.50)		11(18.97)	124(35.03)		17(24.64)	118(34.40)	
Quite agree	131(31.80)	17(24.29)	114(33.33)		18(31.03)	113(31.92)		20(28.99)	111(32.36)	
Totally agree	85(20.63)	26(22.86)	69(20.18)		13(22.41)	72(20.34)		16(23.19)	69(20.12)	
I am well informed about the transmission routes of COVID-19				0.388			0.499			0.254
Totally disagree	4(0.97)	2(2.86)	2(0.58)		2(3.45)	2(0.56)		2(2.90)	2(0.58)	
Not quite agree	6(1.46)	3(4.29)	3(0.88)		2(3.45)	4(1.13)		1(1.45)	5(1.46)	
Average	57(13.83)	9(12.86)	48(14.04)		5(8.62)	52(14.69)		4(5.80)	53(14.45)	
Quite agree	180(43.69)	26(37.14)	154(45.03)		26(44.83)	154(43.50)		29(42.03)	151(44.02)	
Totally agree	165(40.05)	30(42.86)	135(39.47)		23(39.66)	142(40.11)		33(47.83)	132(38.48)	
I am well informed about the infectiousness of COVID-19				0.781			0.605			**0.081**
Totally disagree	5(1.21)	2(2.86)	3(0.88)		2(3.45)	3(0.85)		2(2.90)	3(0.87)	

Not quite agree	6(1.46)	1(1.43)	5(1.46)		1(1.72)	5(1.41)		1(1.45)	5(1.46)	
Average	61(14.84)	9(12.86)	52(15.20)		7(12.07)	54(15.25)		7(10.14)	54(15.74)	
Quite agree	174(42.23)	26(37.14)	148(43.27)		19(32.76)	155(43.97)		20(28.99)	154(44.90)	
Totally agree	166(40.29)	32(45.71)	134(39.18)		29(50.00)	137(38.70)		39(56.52)	127(37.03)	
I am well informed about the effectiveness of preventive measures for COVID-19				0.943			0.246			0.947
Totally disagree	4(0.97)	2(2.86)	2(0.58)		2(3.45)	2(0.56)		2(2.90)	2(0.58)	
Not quite agree	3(0.73)	1(1.43)	2(0.58)		1(1.72)	2(0.56)		1(1.45)	2(0.58)	
Average	63(15.29)	10(14.29)	53(15.50)		10(17.24)	53(14.97)		11(15.94)	52(15.16)	
Quite agree	187(45.39)	26(37.14)	161(47.08)		23(39.66)	164(46.33)		24(34.78)	163(47.52)	
Totally agree	155(37.62)	31(44.29)	124(36.26)		22(37.93)	133(37.57)		31(44.93)	124(36.15)	
I am well informed about the effectiveness of cure of COVID-19				0.327			**<0.001**			**0.040**
Totally disagree	18(4.37)	9(12.86)	9(2.63)		10(17.24)	8(2.26)		8(11.59)	10(2.92)	
Not quite agree	32(7.77)	7(10.00)	25(7.31)		7(12.07)	25(7.06)		5(7.25)	27(7.87)	
Average	141(34.22)	16(22.86)	125(36.55)		17(29.31)	124(35.03)		24(34.78)	117(34.11)	
Quite agree	134(32.52)	18(25.71)	116(33.92)		12(20.69)	122(34.46)		19(27.54)	115(33.53)	
Totally agree	87(21.12)	20(28.57)	67(19.59)		12(20.69)	75(21.19)		13(18.84)	74(21.57)	
I am well informed about the reinfection of COVID-19 after cure				0.359			**0.095**			0.287
Totally disagree	16(3.88)	6(8.57)	10(2.92)		6(10.34)	10(2.82)		5(7.25)	11(3.21)	
Not quite agree	42(10.19)	8(11.43)	34(9.94)		12(20.69)	30(8.47)		9(13.04)	33(9.62)	
Average	165(40.05)	16(22.86)	149(43.57)		15(25.86)	150(42.37)		26(37.68)	139(40.52)	
Quite agree	111(26.94)	20(28.57)	91(26.61)		11(18.97)	100(28.25)		15(21.74)	96(27.99)	
Totally agree	78(18.93)	20(28.57)	58(16.96)		14(24.14)	64(18.08)		14(20.29)	64(18.66)	
**COVID-19 related attitudes [b]**										
I think COVID-19 is very contagious				0.567			0.984			**0.014**
Totally disagree	3(0.73)	1(1.43)	2(0.58)		1(1.72)	2(0.56)		1(1.45)	2(0.58)	
Not quite agree	3(0.73)	0(0.00)	3(0.88)		0(0.00)	3(0.85)		0(0.00)	3(0.87)	
Average	17(4.13)	3(4.29)	14(4.09)		3(5.17)	14(3.95)		0(0.00)	17(4.96)	
Quite agree	58(14.08)	12(17.14)	46(13.45)		6(10.34)	52(14.69)		3(4.35)	55(16.03)	
Totally agree	331(80.34)	54(77.14)	277(80.99)		48(82.76)	283(79.94)		65(94.20)	266(77.55)	

I think COVID-19 is very prevalent where I live				0.213			0.110			0.215
Totally disagree	77(18.69)	15(21.43)	62(18.13)		12(20.69)	65(18.36)		13(18.84)	64(18.66)	
Not quite agree	105(25.49)	10(14.29)	95(27.78)		7(12.07)	98(27.68)		16(23.19)	89(25.95)	
Average	105(25.49)	21(30.00)	84(24.56)		17(29.31)	88(24.86)		14(20.29)	91(26.53)	
Quite agree	44(10.68)	4(5.71)	40(11.70)		5(8.62)	39(11.02)		6(8.70)	38(11.08)	
Totally agree	81(19.66)	20(28.57)	61(17.84)		17(29.31)	64(18.08)		20(28.99)	61(17.78)	
I am afraid of COVID-19				**0.014**			**0.029**			**<0.001**
Totally disagree	42(10.19)	4(5.71)	38(11.11)		4(6.90)	38(10.73)		3(4.35)	39(11.37)	
Not quite agree	52(12.62)	6(8.57)	46(13.45)		6(10.34)	46(12.99)		3(4.35)	49(14.29)	
Average	174(42.23)	27(38.57)	147(42.98)		20(34.48)	154(43.50)		21(30.43)	153(44.61)	
Quite agree	74(17.96)	17(24.29)	57(16.67)		13(22.41)	61(17.23)		17(24.64)	57(16.62)	
Totally agree	70(16.99)	16(22.86)	54(15.79)		15(25.86)	55(15.54)		25(36.23)	45(13.12)	
I think COVID-19 is very close to me				**0.013**			**0.030**			**<0.001**
Totally disagree	19(4.61)	1(1.43)	18(5.26)		0(0.00)	19(5.37)		1(1.45)	18(5.25)	
Not quite agree	41(9.95)	4(5.71)	37(10.82)		4(6.90)	37(10.45)		2(2.90)	39(11.37)	
Average	142(34.47)	21(30.00)	121(35.38)		19(32.76)	123(34.75)		15(21.74)	127(37.03)	
Quite agree	106(25.73)	21(30.00)	85(24.85)		16(27.59)	90(25.42)		21(30.43)	85(24.78)	
Totally agree	104(25.24)	23(32.86)	81(23.68)		19(32.76)	85(24.01)		30(43.48)	74(21.57)	
I felt nervous when I went out during the outbreak				**0.002**			**0.016**			**<0.001**
Totally disagree	74(17.96)	8(11.43)	66(19.30)		7(12.07)	67(18.93)		6(8.70)	68(19.83)	
Not quite agree	108(26.21)	15(21.43)	93(27.19)		13(22.41)	95(26.84)		12(17.39)	96(27.99)	
Average	153(37.14)	25(35.71)	128(37.43)		21(36.21)	132(37.29)		23(33.33)	130(37.90)	
Quite agree	43(10.44)	11(15.71)	32(9.36)		8(13.79)	35(9.89)		15(21.74)	28(8.16)	
Totally agree	34(8.25)	11(15.71)	23(6.73)		9(15.52)	25(7.06)		13(18.84)	21(6.12)	
The outbreak is considered to be under effective control				**0.011**			**0.027**			0.336
Totally disagree	7(1.70)	3(4.29)	4(1.17)		4(6.90)	3(0.85)		3(4.35)	4(1.17)	
Not quite agree	12(2.91)	2(2.86)	10(2.92)		2(3.45)	10(2.82)		4(5.80)	8(2.33)	
Average	63(134)	15(21.43)	48(14.04)		11(18.97)	52(14.69)		7(10.14)	56(16.33)	
Quite agree	134(32.52)	25(35.71)	109(31.87)		16(27.59)	118(33.33)		23(33.33)	111(32.36)	

Totally agree	196(47.57)	25(35.71)	171(50.00)		25(43.10)	171(48.31)		32(46.38)	164(47.81)	
I think there will be another small epidemic				**0.003**			**0.019**			**0.020**
Totally disagree	18(4.37)	3(4.29)	15(4.39)		3(5.17)	15(4.24)		4(5.80)	14(4.08)	
Not quite agree	57(13.83)	7(10.00)	50(14.62)		6(10.34)	51(14.41)		6(8.70)	51(14.87)	
Average	96(23.30)	9(12.86)	87(25.44)		7(12.07)	89(35.14)		11(15.94)	85(24.78)	
Quite agree	138(33.50)	21(30.00)	117(34.21)		18(31.03)	120(33.90)		20(28.99)	118(34.40)	
Totally agree	103(25.00)	30(42.86)	73(21.35)		24(41.38)	79(22.32)		28(40.58)	75(21.87)	
I think the outbreak will cause me financial losses				**0.040**			**0.007**			**0.007**
Totally disagree	8(1.94)	2(2.86)	6(1.75)		2(3.45)	6(1.69)		2(2.90)	6(1.75)	
Not quite agree	25(6.07)	3(4.29)	22(6.43)		2(3.45)	23(6.50)		2(2.90)	23(6.71)	
Average	82(19.90)	8(11.43)	74(21.64)		5(8.62)	77(21.75)		8(11.59)	74(21.57)	
Quite agree	91(22.09)	12(17.14)	79(23.10)		7(12.07)	84(23.73)		9(13.04)	82(23.91)	
Totally agree	206(50.00)	45(64.29)	161(47.08)		42(72.41)	164(46.33)		48(69.57)	158(46.06)	
I think COVID-19 can be cured				0.674			**0.089**			0.301
Totally disagree	14(3.40)	4(5.71)	10(2.92)		6(10.34)	8(2.26)		4(5.80)	10(2.92)	
Not quite agree	16(3.88)	3(4.29)	13(3.80)		2(3.45)	14(3.95)		3(4.35)	13(3.79)	
Average	68(16.50)	9(12.86)	59(17.25)		8(13.79)	60(16.95)		11(15.94)	57(16.62)	
Quite agree	111(26.94)	20(28.57)	91(26.61)		16(27.59)	95(26.84)		20(28.99)	91(26.53)	
Totally agree	203(49.27)	34(48.57)	169(49.42)		26(44.83)	177(50.00)		31(44.93)	172(50.15)	
I think it is possible to be reinfected after COVID-19 is cured				**0.015**			0.240			**0.051**
Totally disagree	14(3.40)	1(1.43)	13(3.80)		2(3.45)	12(3.39)		2(2.90)	12(3.50)	
Not quite agree	35(8.50)	5(7.14)	30(8.77)		8(13.79)	27(7.63)		6(8.70)	29(8.45)	
Average	88(21.36)	9(12.86)	79(23.10)		6(10.34)	82(23.16)		8(11.59)	80(23.32)	
Quite agree	119(28.88)	20(28.57)	99(28.95)		11(18.97)	108(30.51)		18(26.09)	101(29.45)	
Totally agree	156(37.86)	35(50.00)	121(35.38)		31(53.45)	125(35.31)		35(50.72)	121(35.28)	
If I get infected, I will not be able to handle daily activities				**0.100**			**0.034**			**<0.001**
Totally disagree	59(14.32)	7(10.00)	52(15.20)		7(12.07)	52(14.69)		6(8.70)	53(15.45)	
Not quite agree	91(22.09)	17(24.29)	74(21.64)		12(20.69)	79(22.32)		10(14.49)	81(23.62)	

Average	98(23.79)	14(20.00)	84(24.56)		9(15.52)	89(25.14)		12(17.39)	86(25.07)	
Quite agree	72(17.48)	8(11.43)	64(18.71)		7(12.07)	65(18.36)		10(14.49)	62(18.08)	
Totally agree	92(22.33)	24(34.29)	68(19.88)		23(39.66)	69(19.49)		31(44.93)	61(17.78)	
I am very worried about another outbreak				**0.013**			**<0.001**			**<0.001**
Totally disagree	16(3.88)	2(2.86)	14(4.09)		1(1.72)	15(4.24)		1(1.45)	15(4.37)	
Not quite agree	36(8.74)	2(2.86)	34(9.94)		1(1.72)	35(9.89)		1(1.45)	35(10.20)	
Average	107(25.97)	15(21.43)	92(26.90)		9(15.52)	98(27.68)		8(11.59)	99(28.86)	
Quite agree	95(23.06)	16(22.86)	79(23.10)		14(24.14)	81(22.88)		14(20.29)	81(23.62)	
Totally agree	158(38.35)	35(50.00)	123(35.96)		33(56.90)	125(35.31)		45(65.22)	113(32.94)	
**COVID-19 related behavior** [a]										
Had a COVID-19 test				0.937			0.617			0.851
Yes	81(19.66)	14(20.00)	67(19.59)		10(17.24)	71(20.06)		13(18.84)	68(19.83)	
No	331(80.34)	56(80.00)	275(80.41)		48(82.76)	283(79.94)		56(81.16)	275(80.17)	
**Risk perception**										
**Controllability** [b]										
I can take protective measures such as home isolation, wearing masks and washing hands regularly				**0.057**			0.219			0.839
Totally disagree	0(0.00)	0(0.00)	0(0.00)		0(0.00)	0(0.00)		0(0.00)	0(0.00)	
Not quite agree	2(0.49)	1(1.43)	1(0.29)		1(1.72)	1(0.28)		1(1.45)	1(0.29)	
Average	27(6.55)	6(8.57)	21(6.14)		3(5.17)	24(6.78)		4(5.80)	23(6.71)	
Quite agree	59(14.32)	14(20.00)	45(13.16)		13(22.41)	46(12.99)		8(11.59)	51(14.87)	
Totally agree	324(78.64)	49(70.00)	275(80.41)		41(70.69)	283(79.94)		56(81.16)	268(78.13)	
I can ensure that I am not infected with COVID-19				0.324			0.151			0.200
Totally disagree	0(0.00)	0(0.00)	0(0.00)		0(0.00)	0(0.00)		0(0.00)	0(0.00)	
Not quite agree	8(1.94)	2(2.86)	6(1.75)		2(3.45)	6(1.69)		4(5.80)	4(1.17)	
Average	69(16.75)	15(21.43)	54(15.79)		13(22.41)	56(15.82)		14(20.29)	55(16.03)	
Quite agree	150(36.41)	23(32.86)	127(37.13)		20(34.48)	130(36.72)		20(28.99)	130(37.90)	
Totally agree	185(44.90)	30(42.86)	155(45.32)		23(39.66)	162(45.76)		31(44.93)	154(44.90)	
I can control the loss caused by COVID-19				0.465			**0.002**			0.127
Totally disagree	0(0.00)	0(0.00)	0(0.00)		0(0.00)	0(0.00)		0(0.00)	0(0.00)	

Not quite agree	51(12.83)	11(15.71)	40(11.70)		12(20.69)	39(11.02)		12(17.39)	39(11.37)	
Average	223(54.13)	40(57.14)	183(53.51)		36(62.07)	187(52.82)		39(56.52)	184(53.64)	
Quite agree	59(14.32)	4(5.71)	55(16.08)		5(8.62)	54(15.25)		7(10.14)	52(15.16)	
Totally agree	79(19.17)	15(21.43)	64(18.71)		5(8.62)	74(20.90)		11(15.94)	68(19.83)	
**Possibility** [b]										
I'm very likely to get infected with COVID-19				0.685			0.544			0.696
Totally disagree	0(0.00)	0(0.00)	0(0.00)		0(0.00)	0(0.00)		0(0.00)	0(0.00)	
Not quite agree	193(46.84)	32(45.71)	161(47.08)		29(50.00)	164(46.33)		31(44.93)	162(47.23)	
Average	135(32.77)	23(32.86)	112(32.75)		19(32.76)	116(32.77)		24(34.78)	111(32.26)	
Quite agree	29(7.04)	4(5.71)	25(7.31)		3(5.17)	26(7.34)		3(4.35)	26(7.58)	
Totally agree	55(13.35)	11(15.71)	44(12.87)		7(12.07)	48(13.56)		11(15.94)	44(12.83)	
**Susceptibility** [b]										
I am more likely than others to get COVID-19				**0.015**			**0.018**			**0.003**
Totally disagree	73(17.72)	9(12.86)	64(18.71)		8(13.79)	65(18.36)		9(13.04)	64(18.66)	
Not quite agree	146(35.44)	19(27.14)	127(37.13)		15(25.86)	131(37.01)		18(26.09)	128(37.32)	
Average	136(33.01)	26(37.14)	110(32.16)		22(37.93)	114(32.20)		24(34.78)	112(32.65)	
Quite agree	28(6.80)	10(14.29)	18(5.26)		6(10.34)	22(6.21)		10(14.49)	18(5.25)	
Totally agree	29(7.04)	6(8.57)	23(6.73)		7(12.07)	22(6.21)		8(11.59)	21(6.12)	
**Severity** [b]										
I think COVID-19 is very serious				0.380			0.429			**0.036**
Totally disagree	5(1.21)	1(1.43)	4(1.17)		1(1.72)	4(1.13)		1(1.45)	4(1.17)	
Not quite agree	6(1.46)	2(2.86)	4(1.17)		2(3.45)	4(1.13)		1(1.45)	5(1.46)	
Average	39(9.47)	9(12.86)	30(8.77)		9(15.52)	30(8.47)		6(8.70)	33(9.62)	
Quite agree	93(22.57)	13(18.57)	80(23.39)		6(10.34)	87(24.58)		3(4.35)	90(26.24)	
Totally agree	269(65.29)	45(64.29)	224(65.50)		40(68.97)	229(64.69)		58(84.06)	211(61.52)	
**Epidemic exposure** [a]				**0.001**			**0.007**			**0.018**
Yes	209(50.73)	48(68.57)	161(47.08)		39(67.24)	184(51.98)		44(63.77)	165(48.10)	
No	203(49.27)	22(31.43)	181(52.92)		19(32.76)	170(48.02)		25(36.23)	178(51.90)	

Continued

Variables		Higher risk of anxiety			Higher risk of hypochondria					
	Total n (%)	Yes n (%)	No n (%)	p-value	Yes n (%)	No n (%)	p-value			
**Demographic characteristics [a]**										
Age				**0.074**			0.203			
18~35	248(60.19)	46(67.65)	202(58.72)		49(65.33)	199(59.05)				
35~50	142(34.47)	22(32.35)	120(34.88)		25(33.33)	117(34.72)				
⩾50	22(5.34)	0(0.00)	22(6.40)		1(1.33)	21(6.23)				
Household location				**0.093**			0.456			
Urban	388(94.17)	67(98.53)	321(93.31)		72(96.00)	316(93.77)				
Rural	24(5.83)	1(1.47)	23(6.69)		3(4.00)	21(6.23)				
Ethnicity				0.515			0.965			
Han ethnicity	373(90.53)	63(92.65)	310(90.12)		68(90.67)	305(90.50)				
Minority	39(9.47)	5(7.35)	34(9.88)		7(9.33)	32(9.50)				
Educational level				0.868			0.805			
High school or below	61(14.81)	9(13.24)	52(15.12)		11(14.67)	50(14.84)				
Vocational school	150(36.41)	24(35.29)	126(36.63)		25(33.33)	125(37.09)				
College or above	201(48.79)	35(51.47)	166(48.26)		39(52.00)	162(48.07)				
Employment status				0.368			0.674			
Employed	315(74.29)	48(70.59)	267(77.62)		56(74.67)	259(76.85)				
Unemployed/Retirement	73(17.72)	14(20.59)	59(17.15)		13(17.33)	60(17.80)				
Student	24(5.83)	6(8.82)	218(5.23)		6(8.00)	18(5.34)				
Marital status				0.131			0.132			
Married	68(16.50)	7(10.29)	61(17.73)		8(10.67)	60(17.80)				
Unmarried	344(83.50)	61(89.71)	283(82.27)		67(89.23)	277(82.20)				
Monthly personal income				0.365			0.956			
1000~5000 RMB	201(48.79)	36(52.94)	165(47.97)		36(48.00)	165(48.96)				
5000~10000 RMB	139(33.74)	18(26.47)	121(35.17)		25(33.33)	114(33.83)				
⩾10000 RMB	72(17.48)	14(20.59)	58(16.86)		14(18.67)	58(17.21)				
**HIV-related characteristics [a]**										
Sexual role				**0.059**			0.826			

Mainly “Top”	182(44.17)	27(39.71)	155(45.06)		31(41.33)	151(44.81)				
Both of it	72(17.48)	7(10.29)	65(18.90)		13(17.33)	59(17.51)				
Mainly “Bottom”	158(38.35)	34(50.00)	124(36.05)		31(41.33)	127(37.69)				
Number of male sexual partners in the last month				0.103			0.697			
0	227(55.10)	45(66.18)	182(52.91)		42(56.00)	185(54.90)				
1	123(29.85)	17(25.00)	106(30.81)		24(32.00)	99(29.38)				
⩾2	62(15.05)	6(8.82)	56(16.28)		9(12.00)	53(15.73)				
Self-reported HIV infection status				0.138			0.449			
Positive	38(9.22)	10(14.71)	28(8.14)		7(9.33)	31(9.20)				
Negative	358(86.89)	57(83.82)	301(87.50)		67(89.33)	291(86.35)				
Unknown	16(3.88)	1(1.47)	15(4.36)		1(1.33)	15(4.45)				
**COVID-19 related knowledge** [b]										
I am well informed about the causes of COVID-19				**0.082**			0.487			
Totally disagree	21(5.10)	8(11.76)	13(3.78)		4(5.33)	17(5.04)				
Not quite agree	40(9.71)	11(16.18)	29(8.43)		13(17.33)	27(8.01)				
Average	135(32.77)	16(23.53)	119(34.59)		19(25.33)	116(34.42)				
Quite agree	131(31.80)	17(25.00)	114(33.14)		23(30.67)	108(32.05)				
Totally agree	85(20.63)	16(23.53)	69(20.06)		16(21.33)	69(20.47)				
I am well informed about the transmission routes of COVID-19				0.142			**0.050**			
Totally disagree	4(0.97)	2(2.94)	2(0.58)		2(2.67)	2(0.59)				
Not quite agree	6(1.46)	3(4.41)	3(0.87)		4(5.33)	2(0.59)				
Average	57(13.83)	9(13.24)	48(13.95)		11(14.67)	46(13.65)				
Quite agree	180(43.69)	28(41.18)	152(44.19)		30(40.00)	150(44.51)				
Totally agree	165(40.05)	26(38.24)	139(40.41)		28(37.33)	137(40.65)				
I am well informed about the infectiousness of COVID-19				0.644			**0.084**			
Totally disagree	5(1.21)	2(2.94)	3(0.87)		2(2.67)	3(0.89)				
Not quite agree	6(1.46)	1(1.47)	5(1.45)		1(1.33)	5(1.48)				
Average	61(14.84)	13(19.12)	48(13.95)		17(22.67)	44(13.06)				

Quite agree	174(42.23)	21(30.88)	153(44.48)		27(36.00)	147(43.62)				
Totally agree	166(40.29)	31(45.59)	135(39.24)		28(37.33)	138(40.95)				
I am well informed about the effectiveness of preventive measures for COVID-19				**0.084**			**0.088**			
Totally disagree	4(0.97)	2(2.94)	2(0.58)		2(2.67)	2(0.59)				
Not quite agree	3(0.73)	1(1.47)	2(0.58)		1(1.33)	2(0.59)				
Average	63(15.29)	14(20.59)	49(14.24)		16(21.33)	47(13.95)				
Quite agree	187(45.39)	27(39.71)	160(46.51)		29(38.67)	158(46.88)				
Totally agree	155(37.62)	24(35.29)	131(38.08)		27(36.00)	128(37.98)				
I am well informed about the effectiveness of cure of COVID-19				**0.047**			**0.054**			
Totally disagree	18(4.37)	9(13.24)	9(2.62)		8(10.67)	10(2.97)				
Not quite agree	32(7.77)	6(8.82)	26(7.56)		7(9.33)	25(7.42)				
Average	141(34.22)	20(29.41)	121(35.17)		22(29.33)	119(35.31)				
Quite agree	134(32.52)	18(26.47)	116(33.72)		25(33.33)	109(32.34)				
Totally agree	87(21.12)	15(22.06)	72(20.93)		13(17.33)	74(21.96)				
I am well informed about the reinfection of COVID-19 after cure				0.620			0.915			
Totally disagree	16(3.88)	7(10.29)	9(2.62)		7(9.33)	9(2.67)				
Not quite agree	42(10.19)	9(13.24)	33(9.59)		8(10.67)	34(10.09)				
Average	165(40.05)	19(27.94)	146(42.44)		19(25.33)	146(43.32)				
Quite agree	111(26.94)	15(22.06)	96(27.91)		24(32.00)	87(25.82)				
Totally agree	78(18.93)	18(26.47)	60(17.44)		17(22.67)	61(18.10)				
**COVID-19 related attitudes [b]**										
I think COVID-19 is very contagious				0.893			0.379			
Totally disagree	3(0.73)	1(1.47)	2(0.58)		1(1.33)	2(0.59)				
Not quite agree	3(0.73)	0(0.00)	3(0.87)		1(1.33)	2(0.59)				
Average	17(4.13)	2(2.94)	15(4.36)		3(4.00)	14(4.15)				
Quite agree	58(14.08)	10(14.71)	48(13.95)		12(16.00)	46(13.65)				
Totally agree	331(80.34)	55(80.88)	276(80.23)		58(77.33)	273(81.01)				
I think COVID-19 is very prevalent where I live				**0.001**			**0.017**			

Totally disagree	77(18.69)	9(13.24)	68(19.77)		13(17.33)	64(18.99)				
Not quite agree	105(25.49)	9(13.24)	96(27.91)		13(17.33)	92(27.30)				
Average	105(25.49)	21(30.88)	84(24.42)		15(20.00)	90(26.71)				
Quite agree	44(10.68)	6(8.82)	38(11.05)		13(17.33)	31(9.20)				
Totally agree	81(19.66)	23(33.82)	58(16.86)		21(28.00)	60(17.80)				
I am afraid of COVID-19				**0.006**			0.237			
Totally disagree	42(10.19)	4(5.88)	38(11.05)		6(8.00)	36(10.68)				
Not quite agree	52(12.62)	5(7.35)	47(13.66)		11(14.67)	41(12.17)				
Average	174(42.23)	24(35.29)	150(43.60)		24(32.00)	150(44.51)				
Quite agree	74(17.96)	20(29.41)	54(15.70)		20(26.67)	54(16.02)				
Totally agree	70(16.99)	15(22.06)	55(15.99)		14(18.67)	56(16.62)				
I think COVID-19 is very close to me				**0.002**			**0.036**			
Totally disagree	19(4.61)	1(1.47)	18(5.23)		2(2.67)	17(5.04)				
Not quite agree	41(9.95)	2(2.94)	39(11.34)		7(9.33)	34(10.09)				
Average	142(34.47)	21(30.88)	121(35.17)		20(26.67)	122(36.20)				
Quite agree	106(25.73)	19(27.94)	87(25.29)		20(26.67)	86(25.52)				
Totally agree	104(25.24)	25(36.76)	79(22.97)		26(34.67)	78(23.15)				
I felt nervous when I went out during the outbreak				**<0.001**			**0.001**			
Totally disagree	74(17.96)	5(7.35)	69(20.06)		6(8.00)	68(20.18)				
Not quite agree	108(26.21)	14(20.59)	94(27.33)		18(24.00)	90(26.71)				
Average	153(37.14)	26(38.24)	127(36.92)		30(40.00)	123(36.50)				
Quite agree	43(10.44)	12(17.65)	31(9.01)		10(13.33)	33(9.79)				
Totally agree	34(8.25)	11(16.18)	23(6.69)		11(14.67)	23(6.82)				
The outbreak is considered to be under effective control				**0.003**			**0.002**			
Totally disagree	7(1.70)	3(4.41)	4(1.16)		4(5.33)	3(0.89)				
Not quite agree	12(2.91)	3(4.41)	9(2.62)		5(6.67)	7(2.08)				
Average	63(134)	16(23.53)	47(13.66)		15(20.00)	48(14.24)				
Quite agree	134(32.52)	21(30.88)	113(32.85)		20(26.67)	114(33.83)				
Totally agree	196(47.57)	25(36.76)	171(49.71)		31(41.33)	165(48.96)				
I think there will be another small epidemic				**0.029**			**0.065**			

Totally disagree	18(4.37)	3(4.41)	15(4.36)		3(4.00)	15(4.45)				
Not quite agree	57(13.83)	6(8.82)	51(14.83)		6(8.00)	51(15.13)				
Average	96(23.30)	13(19.12)	83(24.13)		18(24.00)	78(23.15)				
Quite agree	138(33.50)	20(29.41)	118(34.30)		22(29.33)	116(34.42)				
Totally agree	103(25.00)	26(38.24)	77(22.38)		26(34.67)	77(22.85)				
I think the outbreak will cause me financial losses				0.269			**0.040**			
Totally disagree	8(1.94)	2(2.94)	6(1.74)		2(2.67)	6(1.78)				
Not quite agree	25(6.07)	1(1.47)	24(6.98)		1(1.33)	24(7.12)				
Average	82(19.90)	12(17.65)	70(20.35)		9(12.00)	73(21.66)				
Quite agree	91(22.09)	16(23.53)	75(21.80)		20(26.67)	71(21.07)				
Totally agree	206(50.00)	37(54.41)	169(49.13)		43(57.33)	163(48.37)				
I think COVID-19 can be cured				**0.098**			**0.066**			
Totally disagree	14(3.40)	5(7.35)	9(2.62)		4(5.33)	10(2.97)				
Not quite agree	16(3.88)	3(4.41)	13(3.78)		5(6.67)	11(3.26)				
Average	68(16.50)	12(17.65)	56(16.28)		14(18.67)	54(16.02)				
Quite agree	111(26.94)	18(26.47)	93(27.03)		20(26.67)	91(27.00)				
Totally agree	203(49.27)	30(44.12)	173(50.29)		32(42.67)	171(50.74)				
I think it is possible to be reinfected after COVID-19 is cured				0.142			**0.050**			
Totally disagree	14(3.40)	1(1.47)	13(3.78)		1(1.33)	13(3.86)				
Not quite agree	35(8.50)	6(8.82)	29(8.43)		6(8.00)	29(8.61)				
Average	88(21.36)	11(16.18)	77(22.38)		8(10.67)	80(23.74)				
Quite agree	119(28.88)	19(27.94)	100(29.07)		28(37.33)	91(27.00)				
Totally agree	156(37.86)	31(45.59)	125(36.34)		32(42.67)	124(36.80)				
If I get infected, I will not be able to handle daily activities				**0.075**			**0.068**			
Totally disagree	59(14.32)	8(11.76)	51(14.83)		8(10.67)	51(15.13)				
Not quite agree	91(22.09)	12(17.65)	79(22.97)		13(17.33)	78(23.15)				
Average	98(23.79)	16(23.53)	82(23.84)		19(25.33)	79(23.44)				
Quite agree	72(17.48)	10(14.71)	62(18.02)		13(17.33)	59(17.51)				
Totally agree	92(22.33)	22(32.35)	70(20.35)		22(29.33)	70(20.77)				

I am very worried about another outbreak				**0.033**			**0.065**			
Totally disagree	16(3.88)	2(2.94)	14(4.07)		2(2.67)	14(4.15)				
Not quite agree	36(8.74)	3(4.41)	33(9.59)		4(5.33)	32(9.50)				
Average	107(25.97)	17(25.00)	90(26.16)		18(24.00)	89(26.41)				
Quite agree	95(23.06)	10(14.71)	85(24.71)		15(20.00)	80(23.74)				
Totally agree	158(38.35)	36(52.94)	122(35.47)		36(48.00)	122(36.20)				
**COVID-19 related behavior [a]**										
Had a COVID-19 test				0.902			0.296			
Yes	81(19.66)	13(19.12)	68(19.77)		18(24.00)	63(18.69)				
No	331(80.34)	55(80.88)	276(80.23)		57(76.00)	274(81.31)				
**Risk perception**										
**Controllability [b]**										
I can take protective measures such as home isolation, wearing masks and washing hands regularly				0.240			0.181			
Totally disagree	0(0.00)	0(0.00)	0(0.00)		0(0.00)	0(0.00)				
Not quite agree	2(0.49)	1(1.47)	1(0.29)		1(1.33)	1(0.30)				
Average	27(6.55)	6(8.82)	21(6.10)		7(9.33)	20(5.93)				
Quite agree	59(14.32)	10(14.71)	49(14.24)		11(14.67)	48(14.24)				
Totally agree	324(78.64)	51(75.00)	273(79.36)		56(74.67)	268(79.53)				
I can ensure that I am not infected with COVID-19				**0.037**			**0.073**			
Totally disagree	0(0.00)	0(0.00)	0(0.00)		0(0.00)	0(0.00)				
Not quite agree	8(1.94)	2(2.94)	6(1.74)		4(5.33)	4(1.19)				
Average	69(16.75)	18(26.47)	51(14.83)		16(21.33)	53(15.73)				
Quite agree	150(36.41)	22(32.35)	128(37.21)		24(32.00)	126(37.79)				
Totally agree	185(44.90)	26(38.24)	159(46.22)		31(41.33)	154(45.70)				
I can control the loss caused by COVID-19				0.532			0.395			
Totally disagree	0(0.00)	0(0.00)	0(0.00)		0(0.00)	0(0.00)				
Not quite agree	51(12.83)	8(11.76)	43(12.50)		11(14.67)	40(11.87)				
Average	223(54.13)	40(58.82)	183(53.20)		42(56.00)	181(53.71)				
Quite agree	59(14.32)	9(13.24)	50(14.53)		9(12.00)	50(14.84)				

Totally agree	79(19.17)	11(16.18)	68(19.77)		13(17.33)	66(19.58)				
**Possibility [b]**										
I'm very likely to get infected with COVID-19				0.906			0.725			
Totally disagree	0(0.00)	0(0.00)	0(0.00)		0(0.00)	0(0.00)				
Not quite agree	193(46.84)	30(44.12)	163(47.38)		31(41.33)	162(48.07)				
Average	135(32.77)	25(36.76)	110(31.98)		29(38.67)	106(31.45)				
Quite agree	29(7.04)	4(5.88)	25(7.27)		6(8.00)	23(6.82)				
Totally agree	55(13.35)	9(13.24)	46(13.37)		9(12.00)	46(13.65)				
**Susceptibility [b]**										
I am more likely than others to get COVID-19				**0.002**			**0.005**			
Totally disagree	73(17.72)	8(11.76)	65(18.90)		10(13.33)	63(18.69)				
Not quite agree	146(35.44)	16(23.53)	130(37.79)		15(20.00)	131(38.87)				
Average	136(33.01)	27(39.71)	109(31.69)		33(44.00)	103(30.56)				
Quite agree	28(6.80)	11(16.18)	17(4.94)		13(17.33)	15(4.45)				
Totally agree	29(7.04)	6(8.82)	23(6.69)		4(5.33)	25(7.42)				
**Severity [b]**										
I think COVID-19 is very serious				0.466			**0.030**			
Totally disagree	5(1.21)	1(1.47)	4(1.16)		2(2.67)	3(0.89)				
Not quite agree	6(1.46)	2(2.94)	4(1.16)		2(2.67)	4(1.19)				
Average	39(9.47)	8(11.76)	31(9.01)		10(13.33)	29(8.61)				
Quite agree	93(22.57)	13(19.12)	80(23.26)		18(24.00)	75(22.26)				
Totally agree	269(65.29)	44(64.71)	225(65.41)		43(57.33)	226(67.06)				
**Epidemic exposure [a]**				**0.024**			**0.022**			
Yes	209(50.73)	43(63.24)	166(48.26)		47(62.67)	162(48.07)				
No	203(49.27)	25(36.76)	178(51.74)		28(37.33)	175(61.93)				

a: Chi-Square Test

b: Trend Chi-Square Test
